# Supplementary material for: Prospective comparative analysis of noninvasive body temperature monitoring using zero heat flux technology (SpotOn sensor) compared with esophageal temperature monitoring during pediatric surgery
Source: PLoS One. 2022 Aug 8;17(8):e0272720. doi: 10.1371/journal.pone.0272720 (PMC9359523; doi:10.1371/journal.pone.0272720)
Supplement: Attachment — Submitted filename: Response to Reviewers.docx [file pone.0272720.s001.docx]

**Response: PONE-D-22-17177, entitled “Prospective comparative analysis of noninvasive body temperature monitoring using zero heat flux technology (SpotOn sensor) compared with esophageal temperature monitoring during pediatric surgery” to PLOS ONE**

Dear Editor-in-Chief Emily Chenette

We would like to thank the editors and reviewers for granting us the possibility to resubmit our manuscript and for their thoughtful reviews. We appreciate truly their time and efforts on our manuscript. Based on reviewers’ advices and suggestions, we have revised the manuscript. Below, we respond to their remarks (in **bold**) and include direct quotes from the manuscript (in *italics*) point-by-point.

Best regards,

Bo-Hyun Sang, MD, PhD

Corresponding author:

Department of Anesthesiology and Pain Medicine, CHA Bundang Medical Center, CHA University, 59 Yatap-ro, Bundang-gu, Seongnam-si, Gyeonggi-do, 13520, Korea. <Tel:82-780-5157> perhaps00@hanmail.net

**EDITOR’S COMMENTS:**

**Journal Requirements:**

**When submitting your revision, we need you to address these additional requirements.**

**1. Please ensure that your manuscript meets PLOS ONE's style requirements, including those for file naming. The PLOS ONE style templates can be found at** [**https://journals.plos.org/plosone/s/file?id=wjVg/PLOSOne_formatting_sample_main_body.pdf and**](https://journals.plos.org/plosone/s/file?id=wjVg/PLOSOne_formatting_sample_main_body.pdf%20and) [**https://journals.plos.org/plosone/s/file?id=ba62/PLOSOne_formatting_sample_title_authors_affiliations.pdf**](https://journals.plos.org/plosone/s/file?id=ba62/PLOSOne_formatting_sample_title_authors_affiliations.pdf)

Thank you for your comment. We checked that our manuscript meets PLOS ONE’s style requirements and revised our manuscript according to PLOS ONE’s style requirements.

**2. Please review your reference list to ensure that it is complete and correct. If you have cited papers that have been retracted, please include the rationale for doing so in the manuscript text, or remove these references and replace them with relevant current references. Any changes to the reference list should be mentioned in the rebuttal letter that accompanies your revised manuscript. If you need to cite a retracted article, indicate the article’s retracted status in the References list and also include a citation and full reference for the retraction notice.**
Thank you for your comment. We checked and revised our references.

**Additional Editor Comments:
The submitted manuscript is well executed and will contribute to the body of knowledge. Please address the points outlined by the reviewers in your revision.**

Thank you very much for your compliment. Below, we respond to the reviewers’ remarks (in **bold**) and include direct quotes from the manuscript (in *italics*) point-by-point.

**REVIEWERS’ COMMENTS:**

**Reviewer #1**

**I would like to thank the editor for the opportunity to review the manuscript entitled "Prospective comparative analysis of noninvasive body temperature monitoring using zero heat flux technology (SpotOn sensor) compared with esophageal temperature monitoring during pediatric surgery". The paper is well-written and the study has been well designed and described.**

**Monitoring body temperature in the clinical setting is of great importance and one of the greatest challenge is represented by the need to measure or estimate core body temperature values with as less invasive as possible techniques due to several limitation that can depend on the setting or patient. This study provides a comparison between a non-invasive device (SpotOn) based on the ZHF technology, and a standard-use technology in ICU or surgery, as the esophageal temperature, in a specific population, i.e., pediatric surgery patients.**

**The results suggest a good agreement between the techniques, but some limitations can be also considered as the time needed to reach good agreement values, and some poor readings when temperature is < 36.5**

**Based on these considerations, the manuscript is worth of publication; however, I have few suggestions I would like to make before acceptance.

In general, I suggest to expand the references a little bit, by adding some papers and reviews on this topic.

- Introduction:
Correctly the authors state that few studies are present about the validation of ZHF in pediatric patients (lines 73-74). Can the authors cite here some of the papers that are already present in the literature, or some reviews? (e.g., Lee et al., 2021; Nemeth et al., 2020, Carvalho et al., 2019; Morettini et al., 2020; Conway et al., 2021).**
 Thank you for your comment and we are sorry for not providing some references.

We cited a reference as follows.

**Introduction section:**

*“However, few studies have been conducted in pediatric patients[15].”*

**Reference section:**

*“15.	Carvalho H, Najafi N, Poelaert J. Intra-operative temperature monitoring with cutaneous zero-heat- flux-thermometry in comparison with oesophageal temperature: A prospective study in the paediatric population. Paediatr Anaesth. 2019;29(8):865-71. Epub 2019/04/30. doi: 10.1111/pan.13653. PubMed PMID: 31034706.”*

**- Results:
Results are well presented, although since the authors present the "temperature zones" analysis, it might be interesting to know how many readings were found in each zone for the esophageal probe. Indeed, since it is reported the lower agreements are present when temperature is < 36.5 °C, it might be important to understand how much common such zone can be during such surgery in pediatric patients.**
Thank you for your advice. We totally agree to your comment. According to your comment, we specify percentage of each zone for the esophageal probe. Of the 466 data pairs, the zone of esophageal temperature < 36.5°C consists of 64 data pairs, which accounted for 13.7%. The zone of esophageal temperature 36.5-37.5°C and >37.5°C each accounted for 362 data pairs (77.7%) and 40 data pairs (8.6%). In this study, since the patients were relatively healthy and the exposure of the surgical field was very narrow, the body temperature during surgery tends to rise rather than drop. It is expected that the proportion of the low body temperature section will be higher in the case of vulnerable children in emergency surgery or in the case of wide exposure to the surgical field. We revised the manuscript as follows.

**Result section***: “Of the 466 data pairs, the zone of esophageal temperature < 36.5°C consists of 64 data pairs, which accounted for 13.7%. The zone of esophageal temperature 36.5-37.5°C and >37.5°C each accounted for 362 data pairs (77.7%) and 40 data pairs (8.6%).”*

**- Discussion:
Based on these findings, I would suggest to well highlight the limitations about the validity of the ZHF system (i.e., time after induction + low body temperature), and maybe provide some sort of recommendations for its use (e.g., to wait n minutes before considering the measurement correct, or to be careful when <36.5 °C is expected).**

Thank you for your suggestion. We strongly agree to your advice. We revised the manuscript as follows.

**Discussion section:** *“These results suggest that the accuracy of the ZHF sensor may be limited in the initial stage after induction or when the body temperature is 36.5°C or less. Based on these results, it is necessary to have doubts about the measured value before the ZHF system is stabilized after induction. In addition, it is recommended that caution is required in interpreting the ZHF sensor value in situations in which the body temperature can be less than 36.5°C, that is, in the cases of emergency surgeries that tend to show unstable body temperature, or in the cases of large exposure to the surgical site.”*

**Reviewer #2**

**In the present study, the authors sought to validate the non-invasive “SpotOn” measurement technic for estimating core temperature against esophageal temperature in children undergoing surgery. The authors found exceptionally good agreement of the SpotOn method. My only comment is that I wish the authors had also compared to other acceptable non-invasive methods for measuring core temperature, such as surface temperature over the carotid (Jay et al, 2013, Pediatric Anesthesia; Imani et al, 2016, Anesthesiology and pain medicine). The authors should mention this technic in their intro and discussion, however, SpotOn had better agreement in this study than surface carotid temperature in those other studies, and therefore mentioning these studies should only serve to strengthen the argument for this new technic. Minor comments below. Great job!**
Thank you for your advice. According to your comment, we revised the manuscript as follows.

**Introduction section:** *“Infrared skin temperature measurement of the upper temporal artery and skin temperature measurement over the carotid artery have been suggested as alternatives to measuring core body temperature with less invasive methods. However, these methods seem to have limitations in their clinical use due to insufficient accuracy of core temperature measurement.”*

**Discussion section:** *“The temporal-artery thermometer and skin temperature over carotid artery, which were suggested as less invasive alternatives to measuring core temperature, were insufficiently accurate for clinical application. In comparison with these methods, the correlation and aggrement between the ZHF sensor and the core temperature represented by the esophageal temperature show more reliable results.”*

**P3, L28 – Missing space after period**
Thank you for your comment. We revised the manuscript as follows.

**Abstract section:** “*intraoperatively. Children*”

**P3, L30 – Missing space after period**
Thank you for your comment. We revised the manuscript as follows.

**Abstract section:** *“enrolled. Body”*

**P3, L37 – This should be -0.07, shouldn’t it?**
Thank you for your comment and we are sorry for our mistake. We revised the manuscript as follows.

**Abstract section:** *“-0.07”*

**P6, L108 – Double ref 10**

Thank you for your comment and we are sorry for our mistake. We deleted one reference.

**Reviewer #3**

**The current manuscript compares the SpotOn sensor to esophageal and axillary temperatures during elective pediatric surgery. Collectively, I have little issues with the manuscript and the statistical analysis done. In my opinion, the purpose of the study has been justified and well executed. However, the main concern I have with the findings is the weaker relationship between esophageal temperature and the SpotOn sensor at lower internal temperatures (<36.5C). In my opinion, this should be more thoroughly highlighted in the discussion, especially when the authors allude to emergency patients (whom still need to be investigated) having greater vulnerability to impaired thermoregulatory control.**

Thank you for your comment and we are sorry for insufficient description regarding the weaker relationship between esophageal temperature and the SpotOn sensor at lower body temperature (<36.5°C). We revised the manuscript as follows.

**Discussion section:** *“These results suggest that the accuracy of the ZHF sensor may be limited in the initial stage after induction or when the body temperature is 36.5°C or less. Based on these results, it is necessary to have doubts about the measured value before the ZHF system is stabilized after induction. In addition, it is recommended that caution is required in interpreting the ZHF sensor value in situations in which the body temperature can be less than 36.5°C, that is, in the cases of emergency surgeries that tend to show unstable body temperature, or in the cases of large exposure to the surgical site.”*
